# The Arabidopsis Cys2/His2 zinc finger transcription factor ZAT18 is a positive regulator of plant tolerance to drought stress

**DOI:** 10.1093/jxb/erx157

**Published:** 2017-06-06

**Authors:** Mingzhu Yin, Yanping Wang, Lihua Zhang, Jinzhu Li, Wenli Quan, Li Yang, Qingfeng Wang, Zhulong Chan

**Affiliations:** 1 Key Laboratory of Aquatic Botany and Watershed Ecology, Wuhan Botanical Garden/Sino-Africa Joint Research Center, Chinese Academy of Sciences, Wuhan, Hubei, China; 2 University of Chinese Academy of Sciences, Beijing, China; 3 Key Laboratory of Horticultural Plant Biology, Ministry of Education, College of Horticulture and Forestry Sciences, Huazhong Agricultural University, Wuhan, Hubei, China; 4 Key Laboratory for Quality Control of Characteristic Fruits and Vegetables of Hubei Province, College of Life Science and Technology, Hubei Engineering University, Xiaogan, Hubei, China

**Keywords:** Drought stress, hormone, RNA sequencing, transcriptome analysis, *ZAT18*, zinc finger proteins

## Abstract

Environmental stress poses a global threat to plant growth and reproduction, especially drought stress. Zinc finger proteins comprise a family of transcription factors that play essential roles in response to various abiotic stresses. Here, we found that ZAT18 (At3g53600), a nuclear C2H2 zinc finger protein, was transcriptionally induced by dehydration stress. Overexpression (OE) of *ZAT18* in Arabidopsis improved drought tolerance while mutation of *ZAT18* resulted in decreased plant tolerance to drought stress. *ZAT18* was preferentially expressed in stems, siliques, and vegetative rosette leaves. Subcellular location results revealed that ZAT18 protein was predominantly localized in the nucleus. *ZAT18* OE plants exhibited less leaf water loss, lower content of reactive oxygen species (ROS), higher leaf water content, and higher antioxidant enzyme activities after drought treatment when compared with the wild type (WT). RNA sequencing analysis showed that 423 and 561 genes were transcriptionally modulated by the *ZAT18* transgene before and after drought treatment, respectively. Pathway enrichment analysis indicated that hormone metabolism, stress, and signaling were over-represented in *ZAT18* OE lines. Several stress-responsive genes including *COR47*, *ERD7*, *LEA6*, and *RAS1*, and hormone signaling transduction-related genes including *JAZ7* and *PYL5* were identified as putative target genes of ZAT18. Taken together, ZAT18 functions as a positive regulator and plays a crucial role in the plant response to drought stress.

## Teaser

ZAT18 modulated genes involved in hormone signaling transduction and stress response pathways, resulting in enhanced osmotic stress responses in seed germination and improved drought stress tolerance.

## Introduction

Drought stress has a negative effect on crop yield and plant distribution. Sessile plants have evolved complex mechanisms to sense and cope with drought conditions. During drought tolerance responses, many drought-responsive genes are activated. Transcription factors (TFs) serve as important mediators in the plants response to drought stress via transcriptional regulation of the downstream genes responsible for plant tolerance, including *DREB*, *ERF*, *MYB*, *MYC*, *WRKY*, *bZIP*, and zinc finger TFs (Rohit *et al.*, 2016).

Zinc finger TFs have been classified into C2H2, C2C2, C2HC, C2C2C2C2, and C2HCC2C2 types according to the number and location of the cysteine and histidine residues (Laity *et al.*, 2001). Genome-wide analysis showed that there were 68 and 67 CCCH family zinc finger TF genes in Arabidopsis and rice (Wang *et al.*, 2008), while 176 and 189 C2H2-type zinc finger TFs were identified in these two species (Englbrecht *et al.*, 2004; Agarwal *et al.*, 2007), indicating that the C2H2-type zinc finger TFs were one of the most abundant TFs in eukaryotes (Laity *et al.*, 2001). The functions of zinc finger proteins (ZFPs) have been well characterized in many plants. Studies revealed that zinc finger TFs played key roles during plant growth and development, and a number of zinc finger TFs were involved in plant abiotic and biotic stress responses (Wu *et al.*, 2008; X.M. Liu *et al.*, 2013). In pepper, a C2H2-type zinc finger TF gene, *CaZFP1*, was induced after inoculation with bacterial pathogens and treatments with ethylene and abscisic acid (ABA). Overexpression of *CaZFP1* in Arabidopsis enhanced resistance against infection by *Pseudomonas syringae* and improved tolerance to drought stress (Kim *et al.*, 2004). In *Thellungiella halophila*, the expression level of the *ThZF1* gene increased after salinity and drought treatments, and overexpression of *ThZF1* in the Arabidopsis *azf2* mutant resulted in similar plant growth and reproductive development to that of the wild type (WT) in the presence of salt (Xu *et al.*, 2007). *GsZFP1*, a C2H2-type zinc finger TF gene from *Glycine soja*, was induced by ABA and abiotic stress treatments. Transgenic *GsZFP1* Arabidopsis plants showed increased tolerance to cold and drought stresses through activation of cold stress-responsive genes and ABA biosynthesis-related genes (Luo *et al.*, 2012). Moreover, overexpression of *CgZFP1*, a C2H2 *ZFP* gene from *Chrysanthemum*, increased salt and drought stress tolerance in Arabidopsis. Under salt or drought conditions, genes involved in osmotic adjustment and reactive oxygen specieS (ROS) scavenging showed enhanced expression in *CgZFP1* transgenic Arabidopsis plants (Gao *et al.*, 2012). The detailed functions of several *ZFP* genes in rice were also characterized. The results indicated that *OsZFP* genes were induced by abiotic stress, and overexpression of these genes improved tolerance to a variety of abiotic stresses in rice (Xu *et al.*, 2008; Huang *et al.*, 2009; Zhang *et al.*, 2012, 2014).

It was reported that the C1 family of C2H2-type zinc finger TFs in Arabidopsis were involved in stress response (Kodaira *et al.*, 2011; W.X. Liu *et al.*, 2013). Twenty members of C2H2-type ZFPs belonging to the C1-2i subclass family have been identified in Arabidopsis, namely AZF1, AZF2, AZF3, Zat5, Zat6, Zat7, Zat8, Zat10, Zat11, Zat12, Zat13, Zat14, Zat15, Zat16, Zat17, Zat18 (At3g53600), At5g04390, At1g02040, At2g26940, and At4g04404 (Ciftci-Yilmaz and Mittlar, 2008). *AZF1* and *AZF2* in Arabidopsis negatively regulated ABA-repressive and auxin-inducible genes under abiotic stress conditions (Kodaira *et al.*, 2011). *AZF2* was shown to be a negative regulator of ABA signaling during seed germination (Gabriele *et al.*, 2010). Many studies have reported that *ZAT6* co-ordinated phosphate homeostasis and root development, and mediated salt and osmotic stress responses (Devaiah *et al.*, 2007; Mito *et al.*, 2011; X.M. Liu *et al.*, 2013). Further analysis showed that *ZAT6* modulated salicylic acid (SA)-related genes and *C-REPEAT-BINDING FACTOR* (*CBF*) genes in response to biotic and abiotic stresses, and might be essential for melatonin-mediated freezing stress resistance in Arabidopsis (Shi and Chan, 2014; Shi *et al.*, 2014*a*). Overexpression of *Zat7* resulted in inhibited growth and increased tolerance to salinity stress, while mutation of *Zat7* abolished salinity tolerance without affecting growth suppression (Ciftci-Yilmaz *et al.*, 2007). More interestingly, gain- and loss-of-function mutations of *ZAT10* both functioned as positive regulators of plant tolerance to osmotic and salt stresses (Mittler *et al.*, 2006). *ZAT11* regulated paraquat-induced programmed cell death in *Arabidopsis thaliana* (Muhammad *et al.*, 2013). Recently, overexpression of *ZAT11* was shown to enhance the elongation of primary roots, but reduced resistance to nickel ion (Ni^2+^) (Liu *et al.*, 2014).

To date, the functions of *ZAT18* in plant response to abiotic stress remain unclear. In this study, we found that *ZAT18* positively modulated drought stress tolerance. Overexpression of *ZAT18* improved seed germination after mannitol treatment and drought tolerance after withholding water. Physiological and transcriptomic changes were monitored after drought treatment. The results suggested that *ZAT18* could play a crucial role in plant responses to drought stress.

## Materials and methods

### Plant materials and growth conditions

The *A. thaliana* Columbia-0 (Col-0) ecotypes, and the T-DNA insertion lines of SALK_027144C and SALK_132289C obtained from the Arabidopsis Biological Resource Center were used in this study. Arabidopsis seeds were surface-sterilized with 50% (v/v) bleach with 0.1% (v/v) Triton X-100, and then washed four times with sterile water. After stratification at 4 °C in the dark for 3 d, seeds were sown on Murashige and Skoog (MS) medium containing 3% (w/v) sucrose and incubated in a growth chamber. The growth chamber was controlled at 21–23 °C, 100 μmol photons m^−2^ s^−1^, 60% relative humidity, and 16 h light/8 h dark cycles.

### Constructs and generation of transgenic plants

For the *ZAT18* plasmid construct, the coding sequence of *ZAT18* was amplified using the primers ZAT18-F and ZAT18-R, then the PCR products was inserted into *Xba*I/*Kpn*I sites of the pCAMBIA1305 vector. For the *ProZAT18::GUS* plasmid construct, a fragment of ~2.4 kb of the 5ʹ upstream region of the *AT3G53600* gene was PCR amplified using the primers *ZAT18*Pro-F and *ZAT18* Pro-R, then the PCR products were inserted into *Sal*I/*Xma*I sites of the pBI101 vector. For the *35S::ZAT18-GFP* plasmid construct, the coding sequence of *ZAT18*, in which the termination codon was removed, was amplified using the primers ZAT18-F and ZAT18-R, then the product was cloned into the pGFP vector using the restriction enzymes *Xba*I and *Kpn*I. The resulting vectors were mobilized into *Agrobacterium tumefaciens* GV3101. Transformation of plants was achieved by the floral-dip method. The T_1_ transgenic seeds were selected on MS agar medium containing 50 mg l^–1^ hygromycin. Each T_1_ plant was individually collected. Selected T_2_ plants were propagated and conﬁrmed by quantitative real-time PCR (qRT-PCR) analysis.

### Subcellular localization analysis and β-glucuronidase (GUS) staining

For subcellular localization analysis, mesophyll protoplasts of 4-week-old Col-0 plants and polyethylene glycol-mediated transformation were performed according to the methods described by Yoo *et al.* (2007). Protoplasts were transfected with *35S::ZAT18-GFP* plasmid. Transformed protoplasts were incubated at 22 °C in the dark for 16–24 h to allow accumulation of green fluorescent protein (GFP) or GFP fusion proteins. The fluorescence was examined at 488 nm by a laser scanning confocal microscope (TCS SP8, Leica, Germany). The method of GUS staining is described by Jefferson *et al* (1987). *ProZAT18::GUS* plants after different periods were immersed in 90% acetone for 20 min, and then stained in GUS staining solution [1 mM X-Gluc, 0.1 M phosphate-buffered saline (PBS), pH 7.0; 2 mM potassium ferricyanide, 2 mM potassium ferrocyanide, 10 mM EDTA] at 37 °C overnight. The plants were decolorized by 70% ethanol overnight. Images were taken using an anatomical lens (SZX16 SZX10, Olympus, Japan). The primers are all listed in Supplementary Table S1 at *JXB* online.

### Drought treatment, water loss, and leaf water content measurement

For the drought tolerance test, water was withheld from 7-day-old plants in pots. The plants were re-watered after 21 d drought treatment and survival rates were then measured. To analyze leaf water status, leaf water loss and leaf water content *in vivo* were determined. The detached leaves grown under control conditions were weighed at 1 h intervals for up to 8 h as described by Quan *et al.* (2016). For measurement of leaf water content, the leaf samples were collected at different time points (7, 14, and 21 d) under control and drought stress, and the FW was immediately measured. The DW was quantified after 16 h incubation at 80 °C, and the formula for calculation of leaf water content (LWC) was as follows: LWC (%)=(FW−DW)/FW×100 (Shi *et al.*, 2012).

### Electrolyte leakage (EL), malondialdehyde (MDA) and ROS contents, and measurement of the activities of antioxidant enzymes

For EL analysis, the detached leaves of control and drought-treated plants at different intervals were incubated in 15 ml of deionized water, and shaken at room temperature for 6 h. The *C*_i_ was measured by a conductivity meter (Leici-DDS-307A, Shanghai, China). The detached leaves were boiled for 20 min and the *C*_max_ was determined after cooling to room temperature: relative EL (%)=(*C*_i_/*C*_max_)×100 (Wang *et al.*, 2013). The concentration of H_2_O_2_ was determined as described (Shi *et al.*, 2012). Briefly, 1 ml of the above supernatant was dissolved in 1 ml of 0.1% titanium sulfate in 20% H_2_SO_4_ (v/v) thoroughly for 10 min, and then centrifuged at 12 000 *g* for 10 min at room temperature. The absorbance of the supernatant was measured at 410 nm using the known concentration of H_2_O_2_ as control. For the measurement of MDA, 0.5 g leaf samples were ground using liquid nitrogen, and then extracted in 5% trichloroacetic acid (TCA; w/v). The supernatants were centrifuged at 12 000 *g* for 10 min, then 2 ml of 0.67% thiobarbituric acid (TBA) (w/v) was added. Well mixed solutions were boiled at 100 °C for 30 min, and the absorbance of the supernatant was measured at 450, 532, and 600 nm after centrifugation. The concentration of MDA was calculated as described (Li *et al.*, 2011). The catalase (CAT) and peroxidase (POD) activities in the samples were determined using a CAT Assay Kit (A007-1, Nanjing Jiancheng Bioengineering Institute, China) and a Plant POD Assay Kit (A084-3, Nanjing Jiancheng Bioengineering Institute), respectively, according to the manufacturer’s instructions.

### Quantitative real-time PCR

qRT-PCR was performed with SYBR green fluorescence and used a CFX96TM Real Time System (Bio-Rad, California, USA). The expression levels of target genes were standardized with ubiquitin 10 (*UBQ10*, AT4G05320). The primers used in qRT-PCR were designed using the web tool Integrated DNA Technologies (http://sg.idtdna.com/scitools/Applications/RealTimePCR/) and are listed in Supplementary Tables S3 and S4. Experiments were repeated three times.

### Transcriptomic analysis

Arabidopsis WT and two *ZAT18* overexpression (OE) lines were grown in moist soil for 7 d. Drought stress was imposed by withholding water for 10 d. The rosette leaves of control and drought-treated WT and *ZAT18* OE plants were then collected for RNA isolation. RNA purity and integrity were checked using the NanoPhotometer^®^ spectrophotometer (IMPLEN, CA, USA) and RNA Nano 6000 Assay Kit of the Bioanalyzer 2100 system (Agilent Technologies, CA, USA), respectively. RNA sequencing analysis was performed by the Novogene Corporation (Beijing, China). A total amount of 3 μg of RNA was used for generation of sequencing libraries using the NEBNext^®^ Ultra™ RNA Library Prep Kit for Illumina^®^ (NEB, USA) following the manufacturer’s recommendations, and index codes were added to attribute sequences to each sample. After cluster generation, the library preparations were sequenced on an Illumina Hiseq platform and 125 bp/150 bp paired-end reads were generated. Clean reads were obtained by removing low quality reads, reads containing adaptor, and poly-N from raw data. At the same time, Q20, Q30, and the GC content of the clean data were calculated. The index of the Arabidopsis genome was built using Bowtie v2.2.3, and paired-end clean reads were aligned to the reference genome using TopHat v2.0.12. HTSeq v0.6.1 was used to count the read numbers mapped to each gene. Then the FPKM (fragments per kilobase of transcript sequence per millions base pairs sequenced) of each gene was calculated based on the length of the gene and the read counts mapped to this gene. Differential expression analysis of drought stress versus control conditions was performed using the DESeq R package (1.18.0). The resulting *P*-values were adjusted using the Benjamini and Hochberg’s approach for controlling the false discovery rate. Genes with an adjusted *P*-value ≤0.05 found by DESeq and fold change ≥2 were assigned as differentially expressed. Two biological replicates were used for each sample. The clean data were submitted to the Gene Expression Omnibus (GEO) database with the accession number GSE 93979.

### Gene Ontology (GO) and pathway enrichment analyses

All differentially expressed genes with a *P*-value ≤0.05 and fold change ≥2 were loaded and annotated in the Classification SuperViewer Tool (http://bar.utoronto.ca/ntools/cgi-bin/ntools_classification_ superviewer.cgi) (Provart and Zhu, 2003). Functional categories of every gene and pathway were assigned using MapMan (http://mapman.gabipd.org/web/guest/mapmanstore) as the classification source (Thimm *et al.*, 2004). For GO term enrichment analysis, differentially expressed genes were analyzed through the Classification SuperViewer Tool w/ Bootstrap (http://bar.utoronto.ca/ntools/cgi-bin/ntools_classification_superviewer.cgi), and GO (ftp://ftp.arabidopsis.org/home/tair/Ontologies/Gene_Ontology) was used as the classification source. The normalized frequency (NF) of each functional category was calculated as following: NF=sample frequency of each category in each sample⁄background frequency of each category in the Arabidopsis genome.

### Hierarchical cluster analysis

The data sets of specific genes were imported for hierarchical cluster analysis. An uncentered matrix and complete linkage method were used with the CLUSTER program (http://bonsai.hgc.jp/~mdehoon/software/cluster/software.htm) (de Hoon *et al.*, 2004). Resulting tree figures were displayed using Java Treeview (http://jtreeview.sourceforge.net/) as described by Chan *et al.* (2012).

### Statistical analysis

All of the experiments in this study were repeated three times, and the values presented are means ±SEs. For each independent experiment, the leaf sample extract was derived from the leaves of at least 15 plants. Asterisks above the columns in figures indicate significant differences relative to the WT at *P*≤0.05 (*t*-test).

## Results

### *ZAT18* transcript was induced by abiotic stress treatment

**Fig. 1.**
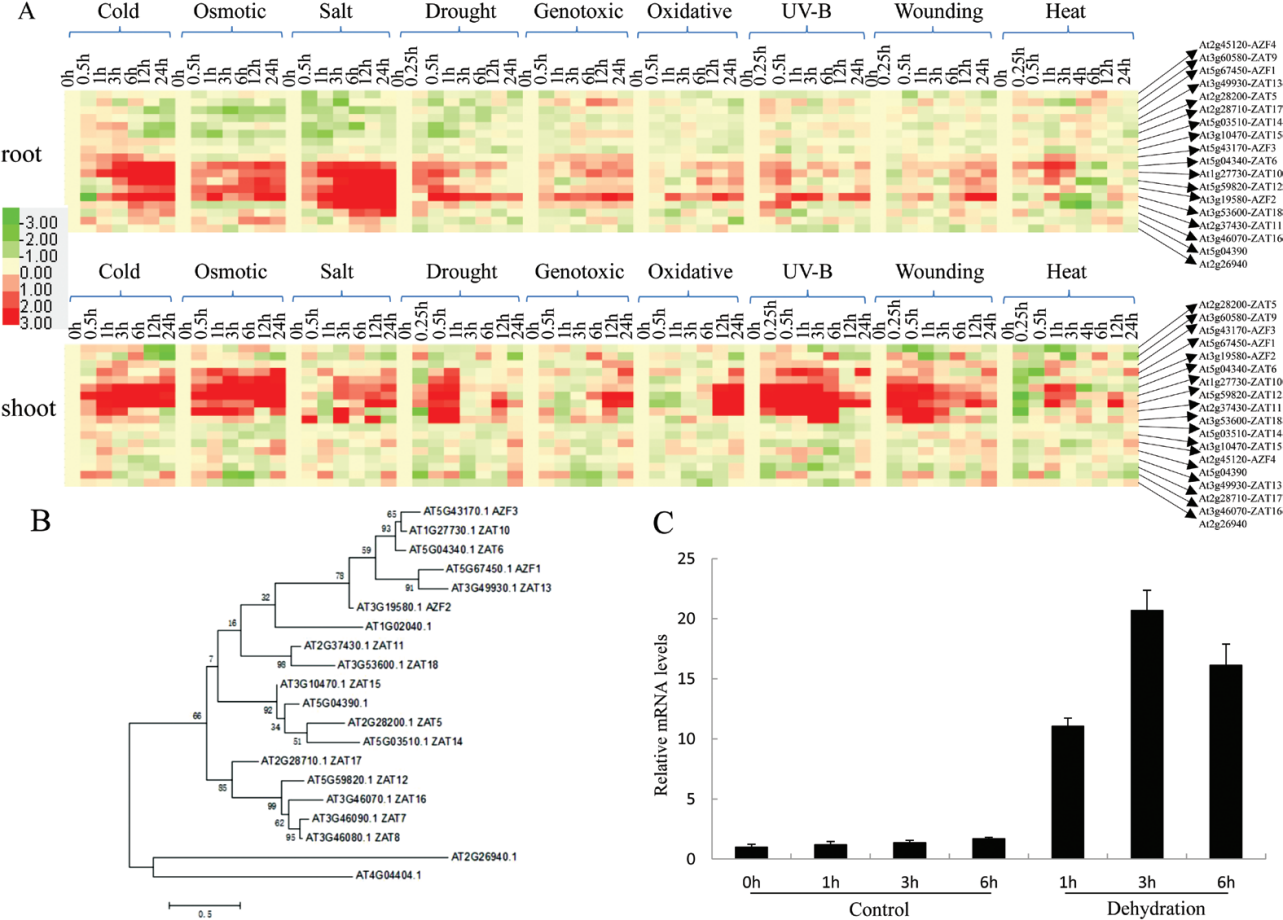
Expression and phylogenetic analysis of the C1-2i subfamily of C2H2 zinc finger transcription factors under abiotic stress conditions. (A) Expression level changes of the C1-2i subfamily of C2H2 zinc finger transcription factors by different abiotic stresses in the root and shoot. The publicly available microarray data were obtained from The Bio-Analytic Resource for Plant Biology (http://bar.utoronto.ca/affydb/cgi-bin/affy_db_exprss_browser_in.cgi). (B) Maximum likelihood tree of C1-2i proteins. (C) Expression of *ZAT18* affected by dehydration through real-time PCR.

Expression level changes of all 20 C1-2i subclass family C2H2 zinc finger TFs in Arabidopsis were analyzed based on publicly available microarray data (Winter *et al.*, 2007). Eighteen genes, with the exception *ZAT7* (At3g46090) and *ZAT8* (At3g46080), were found in these data. The results showed that *AZF2*, *ZAT6*, *ZAT10*, *ZAT12*, *ZAT11*, *ZAT16*, and *ZAT18* (At3g53600) were highly up-regulated especially by cold, osmotic, salt, and drought stresses in root tissue, while *AZF1*, *AZF2*, *ZAT6*, *ZAT10*, *ZAT11*, *ZAT12*, and *ZAT18* were highly induced by cold, osmotic, salt, drought, UV-B, and wounding in shoot tissue (Fig. 1A). In both shoot and root tissue, expression of *ZAT18* was activated by most of the abiotic stress treatments. Phylogenetic analysis showed that *ZAT18* shared high similarity with *ZAT11* (Fig. 1B). qRT-PCR results revealed that the *ZAT18* expression level increased significantly after dehydration treatment, and reached the highest level at 3 h of treatment (Fig. 1C).

**Fig. 2.**
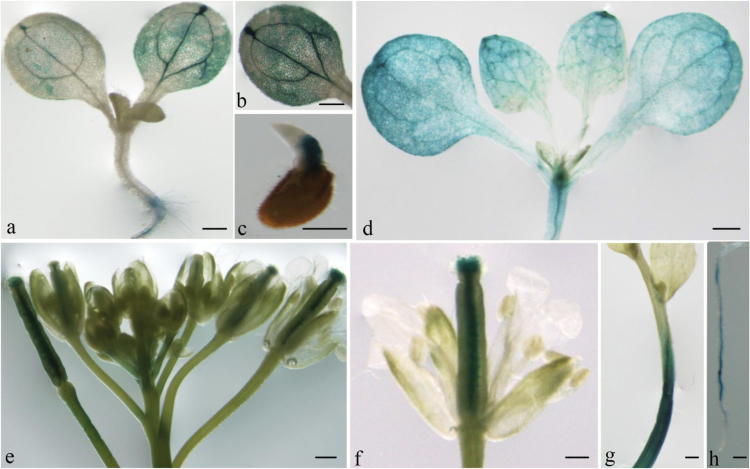
GUS staining of Pro*ZAT18*::GUS transgenic plants in different organs. In the transgenic *ProZAT18::GUS* plants, *ZAT18 was* detected in hypocotyls on the fourth day of seed germination (c), it was partially expressed in leaves, hypocotyls and roots at 8-d-old seedling (a, b, h). *ZAT18* was found to be strongly expressed in leaves and stem apex at 10-d-old seedling (d). During flowering period, *ZAT18* was predominantly expressed in stigmas, styles, siliques and stems (e–g). Scale bar, 1 mm.

**Fig. 3.**
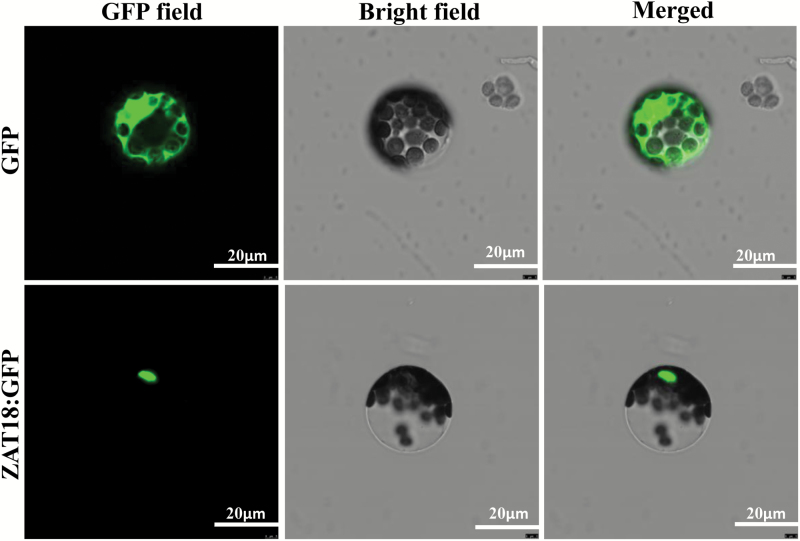
Subcellular location of ZAT18. GFP signals were detected in protoplasts transformed with 35S::*GFP*-*ZAT18*.

In the *ProZAT18::GUS* transgenic plant, *ZAT18* was preferentially expressed in stems, siliques, and vegetative rosette leaves, while lower expression levels could be detected in cotyledons, hypocotyl, and roots (Fig. 2). The subcellular location of ZAT18 was detected. The results revealed that ZAT18–GFP fusion protein fluorescence was predominantly localized in the nucleus. In contrast, the control GFP protein without ZAT18 was distributed in the nucleus and cytoplasm, indicating that ZAT18 was a nuclear-localized protein (Fig. 3).

### Overexpression of *ZAT18* enhanced plant tolerance to drought

**Fig. 4.**
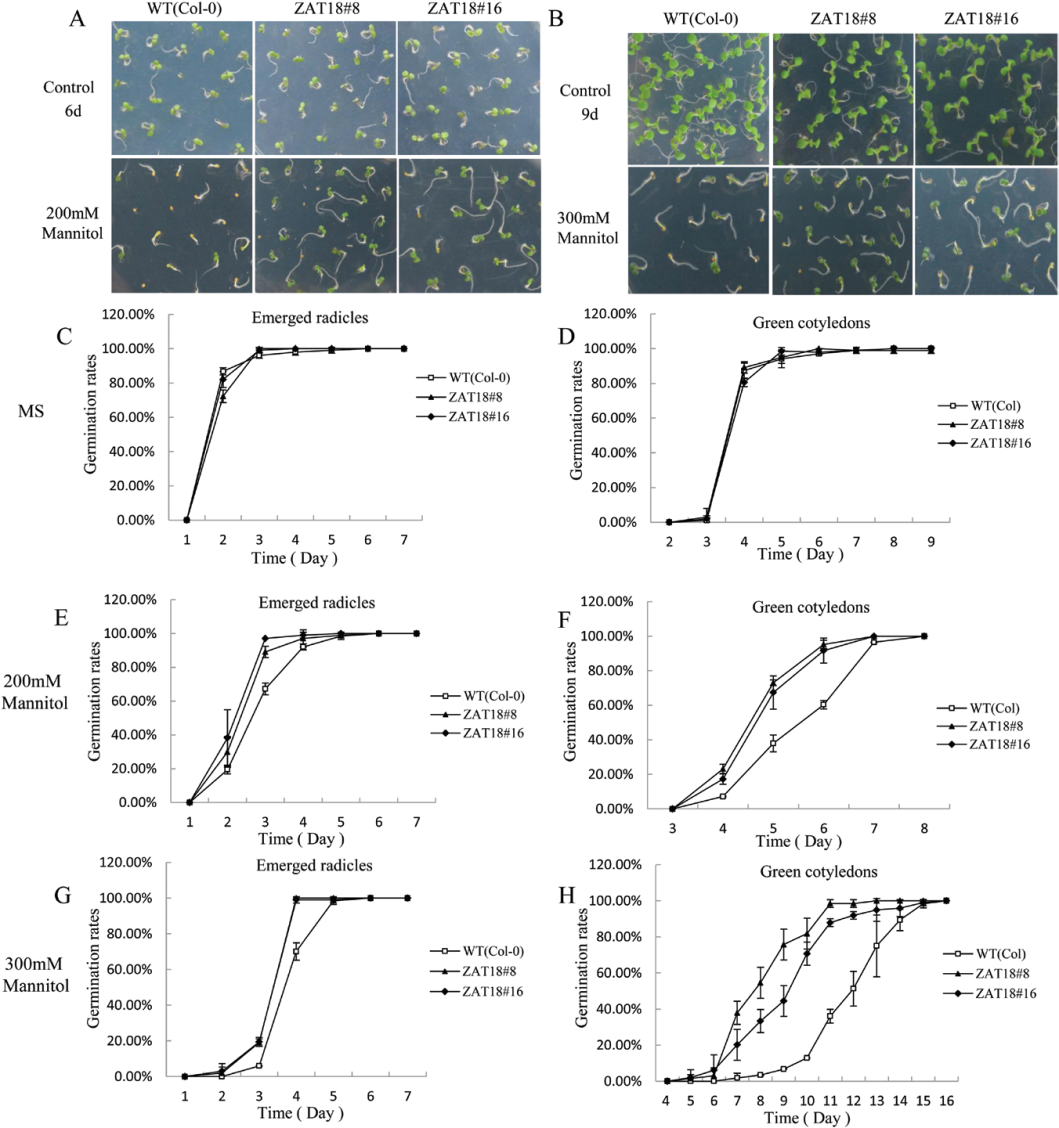
Germination assay of *ZAT18* OE lines under osmotic stress conditions. (A and B) Growth of *ZAT18* OE and WT plants in the presence of 200 mM mannitol for 6 d and 300 mM mannitol for 9 d, respectively. (C and D) Rates of emerged radicles and green cotyledons of *ZAT18* OE and WT plants under control conditions. (E and F) Rates of emerged radicles and green cotyledons of *ZAT18* OE and WT plants after treatment with 200 mM mannitol. (G and H) Rates of emerged radicles and green cotyledons of *ZAT18* OE and WT plants after treatment with 300 mM mannitol. (This figure is available in colour at *JXB* online.)

To characterize the function of *ZAT18* in response to drought stress, we generated *ZAT18* OE plants (Supplementary Fig. S1). Germination assay showed that no significant difference was observed between transgenic and WT plants under normal growth conditions (Fig. 4C, D). However, in the presence of 200 mM and 300 mM mannitol, the *ZAT18* OE lines showed less sensitivity than WT plants during the seed germination stage (Fig. 4E–H). On the fourth day after sowing in the presence of 300 mM mannitol, ~65% of WT seeds germinated with emerged radicles, while >95% of *ZAT18* OE seeds germinated with emerged radicles (Fig. 4G). Additionally, the OE lines showed significantly higher percentages of green cotyledons after 200 mM and 300 mM mannitol for 6 d and 9 d, respectively (Fig. 4A, B). These results indicated that *ZAT18* might act as a positive regulator in osmotic stress during seed germination.

**Fig. 5.**
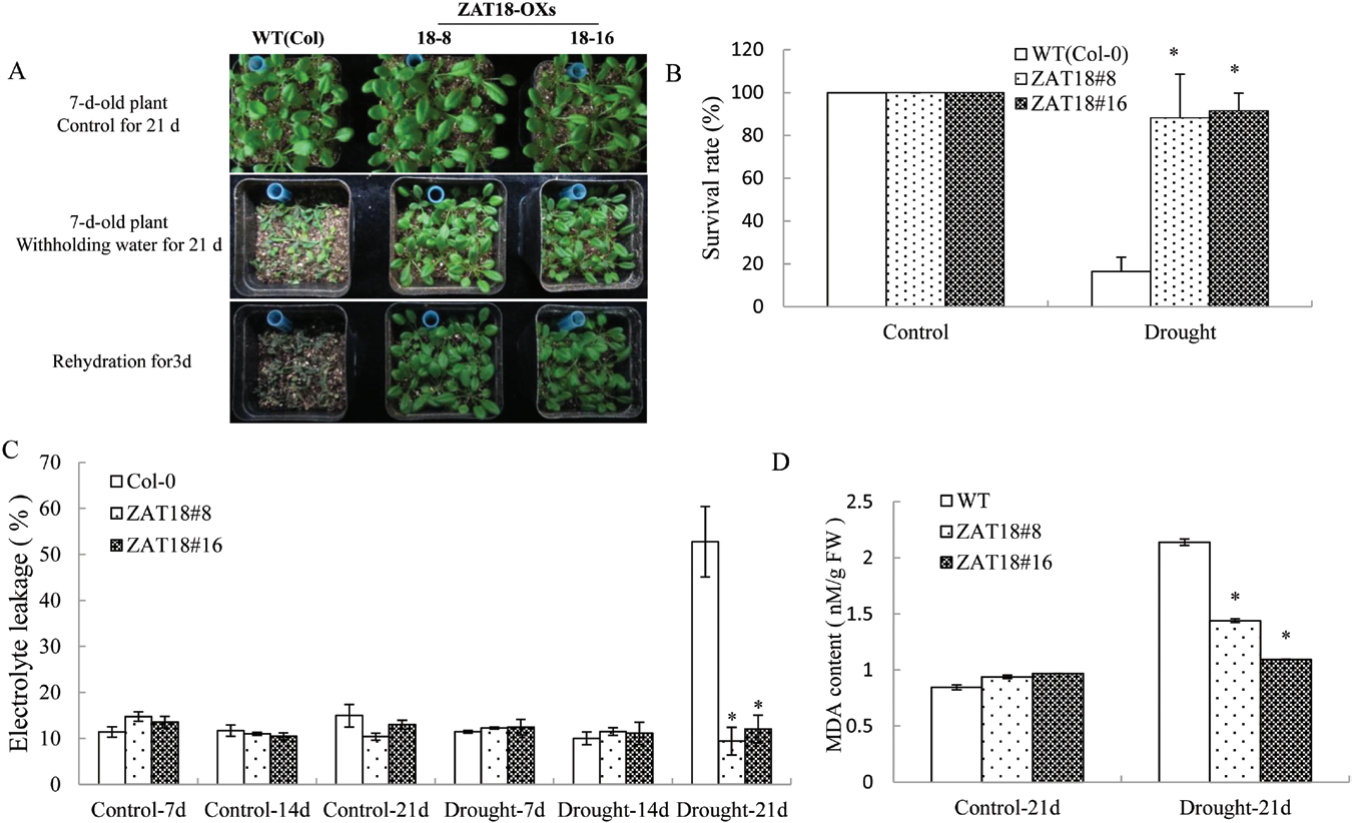
Overexpression of *ZAT18* increased drought stress tolerance in Arabidopsis. (A) Growth of *ZAT18* OE and WT plants after drought treatment and rehydration. (B) Survival rate of *ZAT18* OE and WT plants after drought treatment. (C) Electrolyte leakage of *ZAT18* OE and WT plants. (D) MDA content changes of *ZAT18* OE and WT plants. (This figure is available in colour at *JXB* online.)

**Fig. 6.**
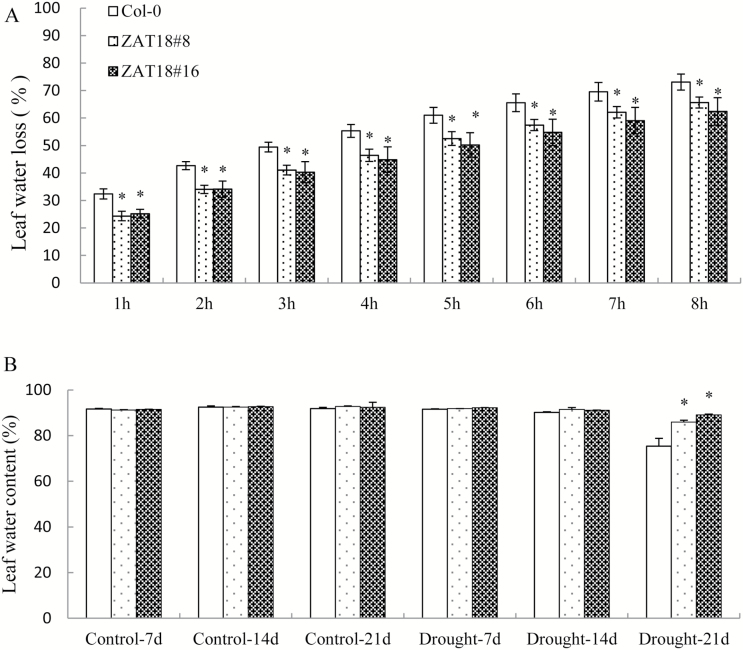
Overexpression of *ZAT18* affected water loss in Arabidopsis. (A) Leaf water loss of *ZAT18* OE and WT plants. (B) Leaf water content of *ZAT18* OE and WT plants after 21 d drought treatment.

We then investigated the drought tolerance of *ZAT18* OE lines. Water was withheld for 3 weeks from 1-week-old WT and OE plants grown under normal conditions, and then they were rehydrated for 3 d. As shown in Fig. 5A, the Col-0 plants showed serious withering and damage after drought treatment, and the survival rate was 16.47% after 3 d rehydration. *ZAT18* OE lines appeared relatively healthy and exhibited high vigor after 3 weeks of drought treatment. The survival rate of *ZAT18* OE lines was ~90% after re-watering (Fig. 5B). The EL did not show significant changes in the control condition between *ZAT18* OE line and the WT. However, 21 d of drought stress caused severe cell membrane damage in WT plants as evidenced by a significantly increased EL, while *ZAT18* OE lines exhibited no significant EL changes (Fig. 5C). The MDA content in *ZAT18* OE plants was significantly lower than in the WT at 21 d after drought treatment (Fig. 5D). Moreover, *ZAT18* OE plants showed significantly lower water loss rates when compared with WT plants (Fig. 6A). The leaf water content of *ZAT18* OE plants was significantly higher than that of Col-0 plants after 3 weeks of withholding water (Fig. 6B). Taken together, these data suggested that the *ZAT18* OE plants showed improved tolerance to severe drought stress.

### Overexpression of *ZAT18* decreased ROS production in response to drought stress

**Fig. 7.**
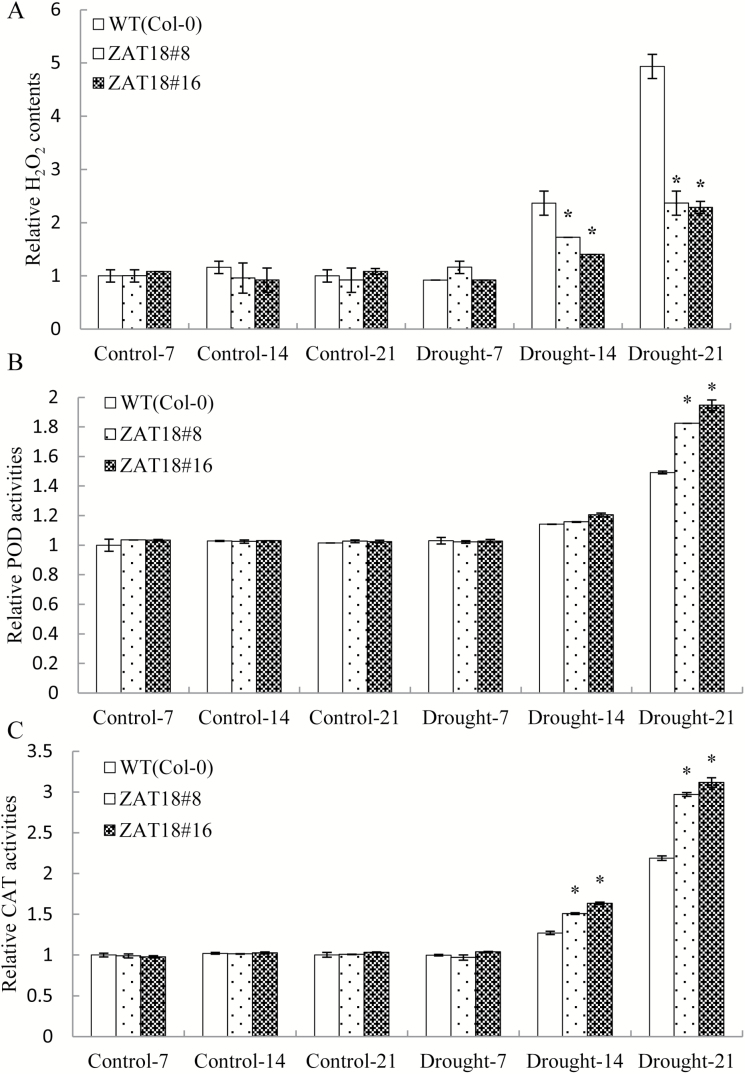
Overexpression of *ZAT18* modulated ROS metabolism in Arabidopsis. (A) H_2_O_2_ content of *ZAT18* OE and WT plants after drought treatment. (B and C) Relative POD and CAT activities of *ZAT18* OE and WT plants after drought treatment.

Drought stress could trigger ROS accumulation in plants, and an efficient ROS detoxification system was essential for plants to survive. In this study, we found that the H_2_O_2_ content of the WT was significantly higher than that in *ZAT18* OE plants after drought treatment for 14 d and 21 d (Fig. 7A). Enzyme activities of CAT and POD were measured after drought stress treatment. The results showed that no significant differences were found for the CAT and POD activities in *ZAT18* OE and WT plants under control and 7 d drought treatment conditions. However, CAT and POD activities in *ZAT18* OE plants were significantly higher than those in Col-0 plants after 14 d and 21 d drought treatment (Fig. 7B, C).

### Mutation of *ZAT18* slightly decreased drought tolerance

**Fig. 8.**
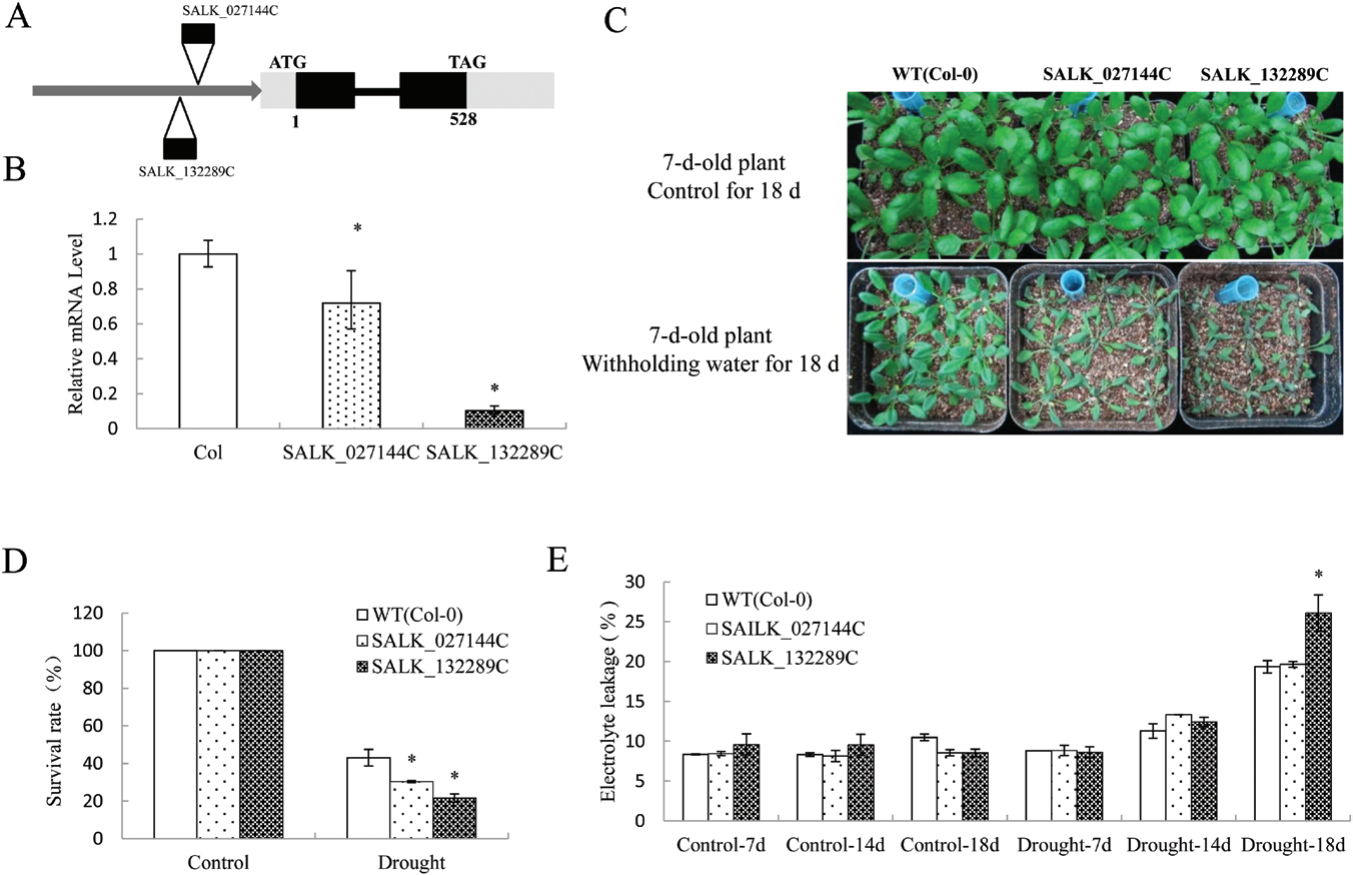
*ZAT18* knock-down mutants were sensitive to drought stress. (A) T-DNA insertion site of two *ZAT18* SALK mutants. (B) Expression level of *ZAT18* in two SALK lines. (C) Drought tolerance test of the *zat18* mutant by withholding water for 18 d. (D) Survival rate of *zat18* mutant and WT plants after drought treatment. (E) Electrolyte leakage of *zat18* mutant and WT plants. (This figure is available in colour at *JXB* online.)

Two *ZAT18* SALK lines were obtained from TAIR (Fig. 8A). Expression analysis indicated that both lines are knock-down lines, and SALK_132289C has a 90% decreased *ZAT18* level (Fig. 8B). Drought tolerance test results showed that both SALK lines were relatively sensitive to drought stress and the survival rates of SALK lines were relatively lower than those of the WT (Fig. 8C, D). EL analysis indicated that SALK_132289C suffered from drought tolerance when compared with the WT and SALK_027144C, as evidenced by a significantly higher EL (Fig. 8E). These results indicated that mutation of *ZAT18* impaired drought stress tolerance.

### Transcriptomic profiling analysis of *ZAT18* OE plants after drought treatment

**Fig. 9.**
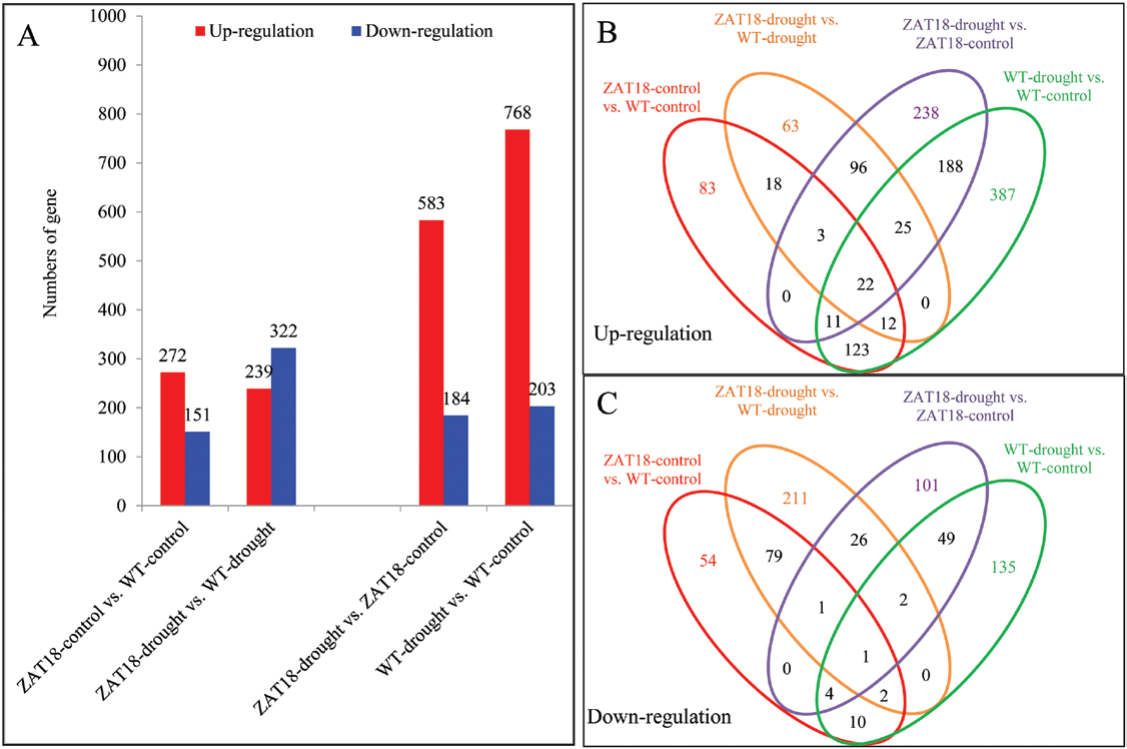
*ZAT18* transgene- and drought stress-modulated genes through RNA-seq analysis. (A) Number of gene affected by the *ZAT18* transgene and drought stress. (B and C) Overlapping analysis of up- and down-regulated genes by *ZAT18* or drought stress. (This figure is available in colour at *JXB* online.)

To dissect *ZAT18*-modulated drought stress tolerance, RNA sequencing (RNA-seq) analysis was performed to identify candidate *ZAT18* target genes. In total, eight samples with two biological replicates per genotype/treatment combination were used for RNA-seq analysis. At least 2 G clean bases were generated for each sample. Comparative analysis revealed that 1777 genes were transcriptionally affected by *ZAT18* transgene or drought treatment (Supplementary Table S2). We selected 11 genes which were modulated by the *ZAT18* transgene and performed qRT-PCR analysis. The expression ratios measured by RNA-seq and by qRT-PCR were highly correlated. The trends of both up-regulated and down-regulated expression for the comparisons were similar (Supplementary Fig. S2). Expression of *ZAT18* (AT3G53600) showed a >60-fold change in *ZAT18* OE lines, indicating that RNA-seq data are at least partially reliable (Supplementary Table S2). The results showed that overexpression of *ZAT18* modulated expression level changes of 423 and 561 genes under control and drought stress conditions, respectively (Fig. 9A). Drought stress treatment changed expression of 971 genes, with 768 up-regulated and 203 down-regulated. Comparatively fewer genes (583 up-regulated and 184 down-regulated) were changed by drought treatment in *ZAT18* OE lines, indicating that several genes were constitutively activated in *ZAT18* OE lines (Fig. 9A). Overlapping analysis indicated that 246 and 56 genes were up- and down-regulated in both *ZAT18* OE lines and the WT after drought treatment (*ZAT18*–drought versus *ZAT18*–control; WT–drought versus WT–control), while 55 and 83 genes were up- and down-regulated by the *ZAT18* transgene before and after drought treatment (*ZAT18*–control versus WT–control; *ZAT18*–drought versus WT–drought) (Fig. 9B, C). These co-regulated genes in *ZAT18* OE lines were the candidate targets of ZAT18 and are listed in Supplementary Table S3.

### Enriched GO terms and pathways

**Fig. 10.**
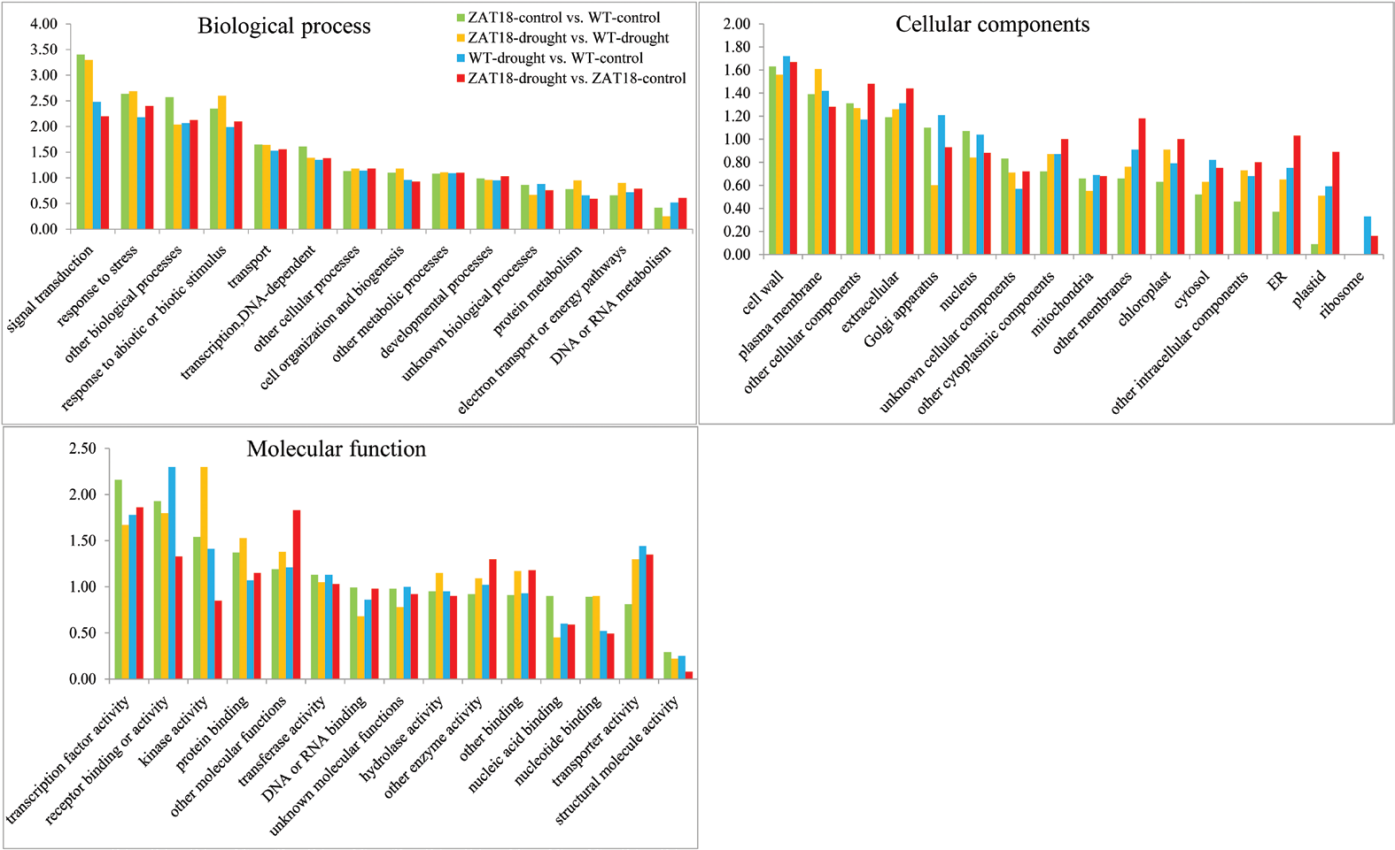
GO term enrichment analysis of genes affected by the *ZAT18* transgene and drought stress treatment. Differentially expressed genes (fold change ≥2 and *P*-value ≤0.05) were annotated using the Classification SuperViewer Tool. GO was used as the classification source. (This figure is available in colour at *JXB* online.)

GO term analysis was performed for genes modulated by the *ZAT18* transgene and drought treatment. For biological process GO terms, signal transduction, response to stress, other biological processes, and response to abiotic or biotic stimulus were enriched. For cellular components GO terms, cell wall, plasma membrane, other cellular components, and extracellular were enriched. For molecular function GO terms, transcription factor activity, receptor binding or activity, and kinase activity were enriched (Fig. 10). Moreover, pathways affected by the *ZAT18* transgene and drought treatment were analyzed. The results showed that five pathways were over-represented for genes affected by both *ZAT18* transgene and drought treatment, including hormone metabolism, miscellaneous, stress, RNA, and signaling. Interestingly, pathways of metal handling and biodegradation of xenobiotics were enriched after drought treatment, but not by the *ZAT18* transgene (Table 1).

**Table 1.**
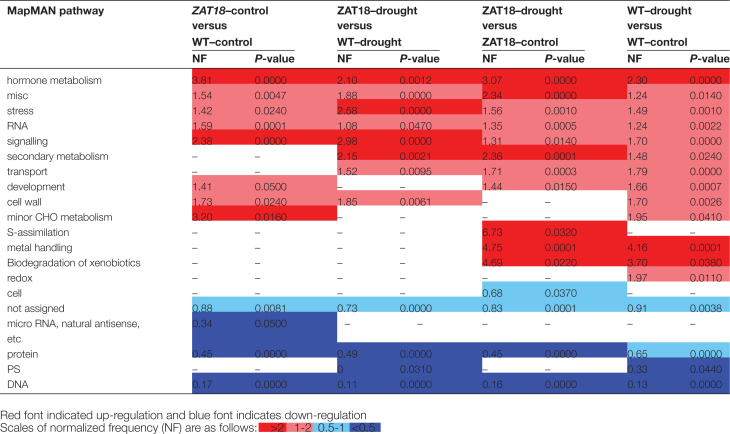
MapMAN pathway enrichment analysis of genes affected by *ZAT18* transgene and drought stress treatment Differentially expressed genes (fold change ≥2 and *P*-value ≤0.05) were annotated using the Classification SuperViewer Tool. MapMAN was used as the classification source

### Identification of candidate target genes of ZAT18

**Fig. 11.**
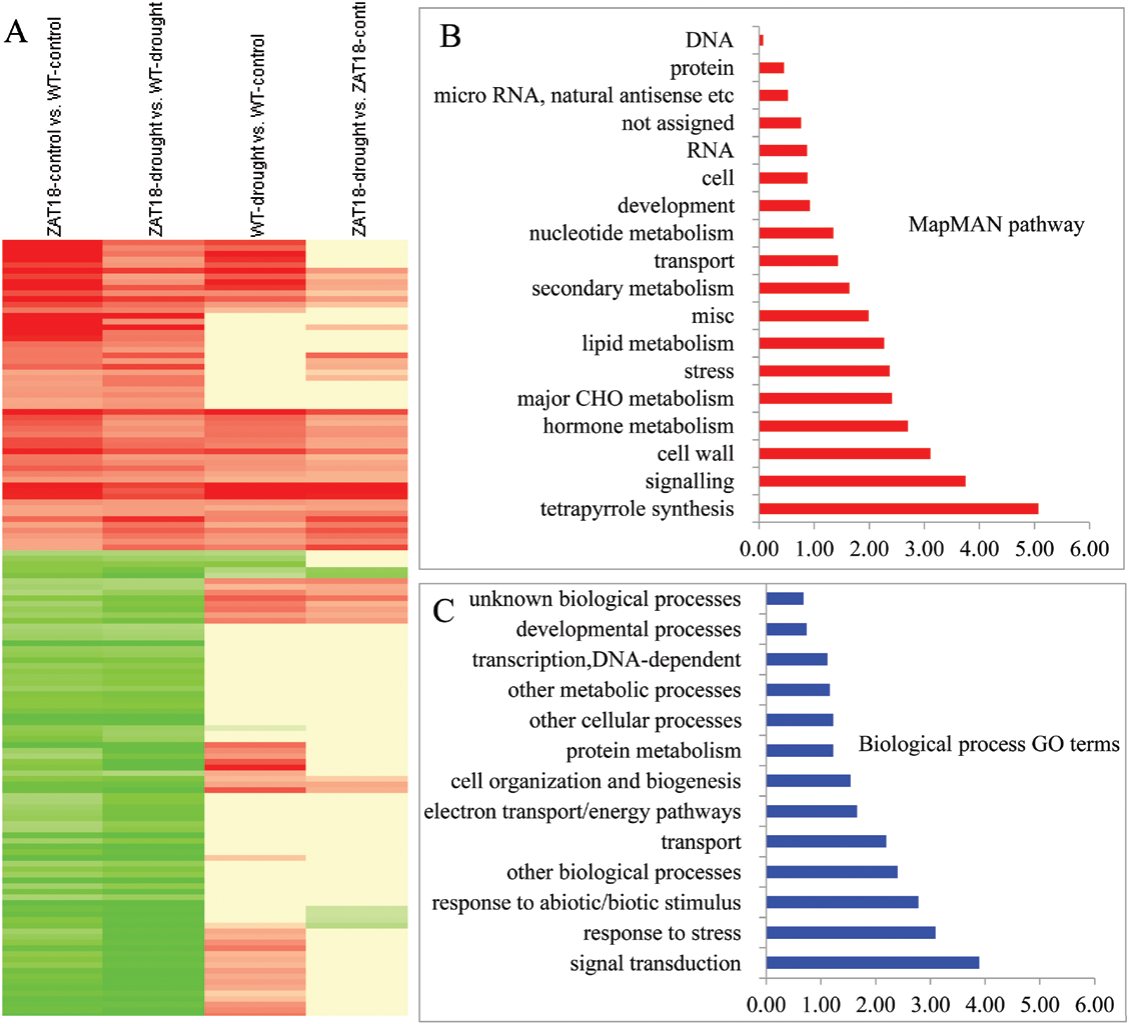
Identification of candidate target genes of *ZAT18*. In total, 138 genes were transcriptionally modulated in *ZAT18* OE lines under both control and drought stress conditions. (A) Cluster analysis of *ZAT18* candidate target genes. (B) Pathway enrichment analysis of *ZAT18* candidate target genes. (C) GO term enrichment analysis of *ZAT18* candidate target genes.

In total, 138 genes were transcriptionally modulated by the *ZAT18* transgene under both control and drought stress conditions (Fig. 11A; Supplementary Table S3). Functional analysis revealed that the enriched pathways included tetrapyrrole synthesis, signaling, cell wall, hormone metabolism, major CHO metabolism, stress, and lipid metabolism (Fig. 11B), and enriched GO terms included signal transduction, response to stress, response to abiotic or biotic stimulus, other biological processes, and transport (Fig. 11C).

Among these candidate genes, 22 of them were up-regulated and 1 of them was down-regulated in all four comparisons. Additionally five drought-inducible genes were down-regulated by the *ZAT18* transgene (Table 2; Fig. 9B, C). Several genes including *RAS1*, *ERD7*, *LEA6*, *COR47*, *ARCK1*, and *At4g35985* were directly involved in plant abiotic stress response. Other genes were key components in hormone signaling transduction including *JAZ7* and *SAUR79*, while *WRKY18*, *NAC081*, *DDF1*, *WRKY70*, and *MYB48* were TF genes which play vital roles in stress response and flavonol biosynthesis (Table 2).

**Table 2.** Genes affected by both the *ZAT18* transgene and drought stress treatment Red font means up-regulation while blue font means down-regulation

AGI	*ZAT18*–control		*ZAT18*–drought		WT–drought		*ZAT18*–drought		Annotation
	versus WT–control		versus WT–drought		versus WT–control		versus *ZAT18*–control		
	log2 FC	*P*-value	log2 FC	*P*-value	log2 FC	*P*-value	log2 FC	*P*-value	
AT1G09950	3.86	0.0003	2.31	0.0000	3.69	0.0000	2.15	0.0000	RAS1_response to ABA and salt 1
AT2G17840	2.12	0.0000	1.69	0.0000	1.43	0.0000	1.01	0.0000	ERD7_Senescence/dehydration-associated protein
AT2G23120	1.03	0.0000	1.31	0.0004	1.11	0.0000	1.40	0.0002	LEA6_Late embryogenesis abundant protein, group 6
AT1G20440	1.15	0.0001	1.59	0.0004	1.21	0.0000	1.65	0.0004	COR47_RD17__cold-regulated 47
AT4G35985	1.72	0.0000	1.31	0.0000	1.61	0.0000	1.21	0.0000	Senescence/dehydration-associated protein-related
AT2G34600	3.21	0.0002	2.26	0.0000	4.54	0.0000	3.60	0.0000	JAZ7_TIFY5B_jasmonate-zim-domain protein 7
AT4G31800	1.76	0.0032	2.54	0.0000	1.43	0.0030	2.21	0.0001	ATWRKY18
AT5G08790	2.76	0.0000	2.10	0.0000	2.28	0.0000	1.64	0.0000	ATAF2_anac081_NAC
AT2G35290	2.12	0.0000	1.37	0.0001	1.80	0.0008	1.06	0.0009	SAUR79_small auxin upregulated RNA 79
AT2G39975	1.56	0.0036	1.16	0.0000	1.64	0.0000	1.26	0.0000	Unknown protein
AT3G16857	1.88	0.0000	1.73	0.0000	1.23	0.0060	1.08	0.0001	ARR1_RR1_response regulator 1
AT5G63450	5.03	0.0000	2.31	0.0000	3.91	0.0000	1.20	0.0000	CYP94B1_cytochrome P450, family 94, subfamily B
AT2G37940	2.12	0.0000	1.62	0.0000	1.52	0.0000	1.03	0.0000	AtIPCS2_Inositol phosphorylceramide synthase 2
AT2G43230	1.60	0.0102	1.34	0.0000	1.26	0.0000	1.01	0.0142	Protein kinase superfamily protein
AT4G20860	1.26	0.0016	1.16	0.0020	1.21	0.0023	1.12	0.0037	FAD-binding Berberine family protein
AT4G34412	2.75	0.0000	1.97	0.0000	3.71	0.0003	2.94	0.0000	Unknown protein
AT1G10260	1.05	0.0099	1.12	0.0000	1.38	0.0000	1.45	0.0000	transposable element gene
AT3G47720	3.05	0.0009	1.23	0.0126	2.83	0.0032	1.02	0.0293	SRO4_similar to RCD one 4
AT1G12610	3.12	0.0000	2.36	0.0000	1.87	0.0008	1.13	0.0000	DDF1_Integrase-type DNA-binding superfamily protein
AT1G15010	2.67	0.0000	2.18	0.0000	3.21	0.0000	2.72	0.0000	Unknown protein
AT1G72520	1.39	0.0000	1.90	0.0000	1.50	0.0006	2.03	0.0000	LOX4_ lipoxygenase 4
AT2G32150	1.03	0.0000	1.74	0.0000	1.49	0.0000	2.22	0.0000	HAD_haloacid dehalogenase-like hydrolase
AT3G56400	–1.66	0.0000	–2.10	0.0000	–1.02	0.00	–1.45	0.0036	WRKY70
AT3G46130	–1.11	0.0042	–1.06	0.0001	1.35	0.0000	1.41	0.0001	ATMYB48
AT2G34940	–1.02	0.0281	–1.42	0.0000	1.41	0.0000	1.02	0.0273	VSR5_vacuolar sorting receptor 5
AT1G76960	–1.73	0.0011	–2.01	0.0000	1.91	0.0000	1.63	0.0061	Unknown protein
AT5G45380	–1.49	0.0181	–2.06	0.0000	1.64	0.0000	1.08	0.0449	ATDUR3_degradation of urea 3
AT4G11890	–1.80	0.0000	–2.16	0.0000	1.48	0.0000	1.13	0.0218	ABA and osmotic stress inducible kinase ARCK1

## Discussion

As transcription repressors, C2H2 ZFPs have been functionally well characterized. As a large gene family, ZFPs play key roles in response to various stresses. Expression of C2H2 zinc finger TFs was induced by different abiotic stresses. *AZF1* and *ZAT10* were strongly induced by NaCl or cold treatment, but weakly by ABA treatment. *AZF2* was induced by NaCl or ABA treatment but weakly by low temperature (Hideki *et al.*, 2000). Overexpression of *ZAT6* enhanced drought, NaCl, and cold tolerance (Shi *et al.*, 2014*a*), whereas overexpression of *ZAT10* conferred salt tolerance (Sakamoto *et al.*, 2004). Bioinformatic analysis showed that expression of *ZAT18* was highly induced by drought stress in both shoot and root tissues (Fig. 1A, C) which prompted us to characterize the roles of *ZAT18* during the plant drought stress response. Overexpression of *ZAT18* conferred improved tolerance to drought stress (Fig. 5A) and mutation of *ZAT18* produced relatively decreased drought tolerance in Arabidopsis. These results indicated that *ZAT18* functioned as a positive drought stress regulator.

Plants respond to drought stress from the cellular to the whole-plant level. Water loss is crucial for plant tolerance to drought stress. In this study, *ZAT18* OE plants had a lower water loss rate and higher leaf water content than the WT under drought stress conditions for 21 d (Fig. 7A, B). The results indicated that *ZAT18* OE lines maintained a higher water status to alleviate water deficiency and showed improved tolerance to drought stress when compared with the WT. The EL is the index of membrane injury. MDA is the final product of lipid peroxidation and its content can reflect stress tolerance of plants. Lower EL and MDA in *ZAT18* OE plants indicated that *ZAT18* OE plants suffered less cell injury than the WT (Fig 6D, E). These results were consistent with the higher survival rate of *ZAT18* OE plants after drought stress treatment (Fig. 6B).

In response to stress, plants activate metabolic processes and accumulate osmolytes that are beneficial to retain water and antioxidants that protect cells from stress-related ROS (Kang *et al.*, 2011). A series of ROS, such as H_2_O_2_ and O^2−^, were produced under stress conditions, resulting in lipid peroxidation, protein oxidation, DNA fragmentation, enzyme inhibition, and cell death (Apel and Hirt, 2004; Wang *et al.*, 2009). Significantly lower H_2_O_2_ levels of *ZAT18* OE plants were observed after 21 d of drought treatment, suggesting that the *ZAT18* OE plants suffered less oxidative damage from drought stress (Fig. 8A). In order to scavenge stress-induced ROS, plants develop a complex antioxidative defense system, which is composed of the non-enzymatic and enzymatic antioxidants. This system is crucially important for plants to survive under severe stress conditions (Mittler, 2002; Foyer and Noctor, 2005; Sharma *et al.*, 2012). As antioxidant enzymes, POD and CAT are responsible for detoxification of H_2_O_2_. Increased enzymes activities would decrease ROS levels (Apel and Hirt, 2004; Ouyang *et al.*, 2010). *ZAT18* OE plants exhibited significantly higher POD and SOD activities after drought treatment (Fig. 8B, C). Transcriptomic analysis showed that the peroxidases *AT5G64120* and *AT3G01420* were highly induced by the *ZAT18* transgene at the gene expression level (Supplementary Table S2).

Among 20 members of the C2H2-type ZFP genes belonging to the C1-2i subclass family, evidence showed that *AZF1*, *AZF2*, *ZAT6*, *ZAT7*, *ZAT10*, *ZAT11*, and *ZAT12* were involved in plant abiotic stress response (Mittler *et al.*, 2006; Ciftci-Yilmaz *et al.*, 2007; Devaiah *et al.*, 2007; Gabriele *et al.*, 2010; Kodaira *et al.*, 2011; Mito *et al.*, 2011; X.M. Liu *et al.*, 2013; Muhammad *et al.*, 2013; Liu *et al.*, 2014; Shi and Chan, 2014; Shi *et al.*, 2014*a*). Previously we determined that *ZAT6* binds directly to the TACAAT motifs in the promoter region of pathogen-related genes and *CBF1* (Shi and Chan, 2014; Shi *et al.*, 2014*a*). However, the target genes of most C2H2-type zinc finger TFs remain elusive. In this study, we identified 423 and 561 genes which showed significant changes in *ZAT18* OE lines before and after drought treatment through RNA-seq analysis (Fig. 9A). Several stress-responsive genes and hormone signaling transduction-related genes were transcriptionally modulated and characterized as putative target genes of *ZAT18*. Among them, *ERD7*, *LEA6*, *COR47*/*RD17*, and *RAS1* were up-regulated by the *ZAT18* transgene. *ERD* genes are a group of plant genes induced by plant stress and ABA (Rai *et al.*, 2016). *LEA* genes are not only highly expressed during late stages of seed development, but are also induced by abiotic stress conditions (Babu *et al.*, 2004; Yu *et al.*, 2016). *COR47* is a cold- and drought-inducible gene which contains a *cis*-acting element called DRE/CRT (for drought/cold-responsive element) which is critical for drought-/cold-induced gene expression (Chinnusamy *et al.*, 2004). CBF3/DREB1A and DREB2A showed 3.3- and 2.6-fold changes in the *ZAT18* OE line under control conditions (Supplementary Table S2). *Response to ABA and Salt 1* (*RAS1*) is an ABA- and salt stress-inducible gene and encoded a previously undescribed plant-specific protein, which required a functional ABA signaling pathway (Ren *et al.*, 2010). Moreover, several genes involved in hormone signaling transduction pathways were identified as candidate *ZAT18* target genes including *JAZ7* and *PYL5*. *JAZ7* is one of the more enigmatic members of the family of 13 JAZ protein genes of Arabidopsis which mediated dark-induced senescence and abiotic stress response (Christine, 2016). *PYL5* is an ABA receptor gene, and overexpression of *PYL5* improved drought stress tolerance and increased antioxidant enzyme activity and osmolyte levels (Shi *et al.*, 2014*b*). Transcriptomic analysis indicated that GO terms including signal transduction, response to stress, and response to abiotic or biotic stress were enriched in *ZAT18* OE lines (Fig. 10). Pathway analysis results showed that hormone metabolism, stress, and signaling were over-represented in *ZAT18* OE lines (Table 1). These results indicated that the *ZAT18* transgene modulated hormone signaling transduction pathways which might activate the downstream stress response.

In conclusion, we partially dissected the functions of *ZAT18* in drought stress responses. *ZAT18* overexpression enhanced osmotic stress responses in seed germination and improved drought stress tolerance. The *ZAT18* transgene increased leaf water content and decreased ROS content. Transcriptomic analysis showed that genes involved in the hormone signaling transduction pathway and stress response were putative targets of *ZAT18*. Further research on the detailed mechanisms of how *ZAT18* modulates the target genes is needed.

## Supplementary data

Supplementary data are available at *JXB* online.

Fig. S1. *ZAT18* expression level in wild-type and *ZAT18* OE plants by qRT-PCR.

Fig. S2. Expression level changes of ZAT18 candidate target genes by qRT-PCR.

Table S1. The primers used for plasmid construction and identification of SALK_027144C and SALK_132289C in this study.

Table S2. List of genes affected by the *ZAT18* transgene and drought stress.

Table S3. List of candidate target genes of ZAT18.

Table S4. The primers of ZAT18 candidate target genes used for for qRT-PCR.

## Supplementary Material

erx157_suppl_supplementary_figuress1_s2_tables1_tables4

erx157_suppl_supplementary_tables2

erx157_suppl_supplementary_tables3

## Abbreviations:

ABA: abscisic acid
CBF: C-REPEAT-BINDING FACTOR
CAT: catalase
DREB: dehydration-responsive element-binding factor
EL: electrolyte leakage
ERF: ethylene-responsive element-binding factor
GO: Gene Ontology
LWC: leaf water content
MDA: malondialdehyde
NF: normalized frequency
JA: jasmonic acid
OE: overexpression
POD: peroxidase
ROS: reactive oxygen species
SA: salicylic acid
TF: transcription factor
WT: wild type
ZFP: zinc finger protein.
