# Supplementary material for: The Arabidopsis Cys2/His2 zinc finger transcription factor ZAT18 is a positive regulator of plant tolerance to drought stress
Source: J Exp Bot. 2017 Jun 6;68(11):2991–3005. doi: 10.1093/jxb/erx157 (PMC5853917; doi:10.1093/jxb/erx157)
Supplement: erx157_suppl_supplementary_figuress1_s2_tables1_tables4 [file erx157_suppl_supplementary_figuress1_s2_tables1_tables4.pdf]

***Arabidopsis* Cys2/His2 zinc-finger transcription factor ZAT18 is a positive regulator of plant tolerance to drought stress.**
Mingzhu Yin, Yanping Wang, Lihua Zhang, Jinzhu Li,Wenli Quan, Li Yang, Qingfeng Wang, Zhulong Chan

Supplementary Fig.1. *ZAT18* expression levels in wild-type and *ZAT18* OE plants. The expression level of *ZAT18* in the wild type was normalized as 1.0 (n = 3).

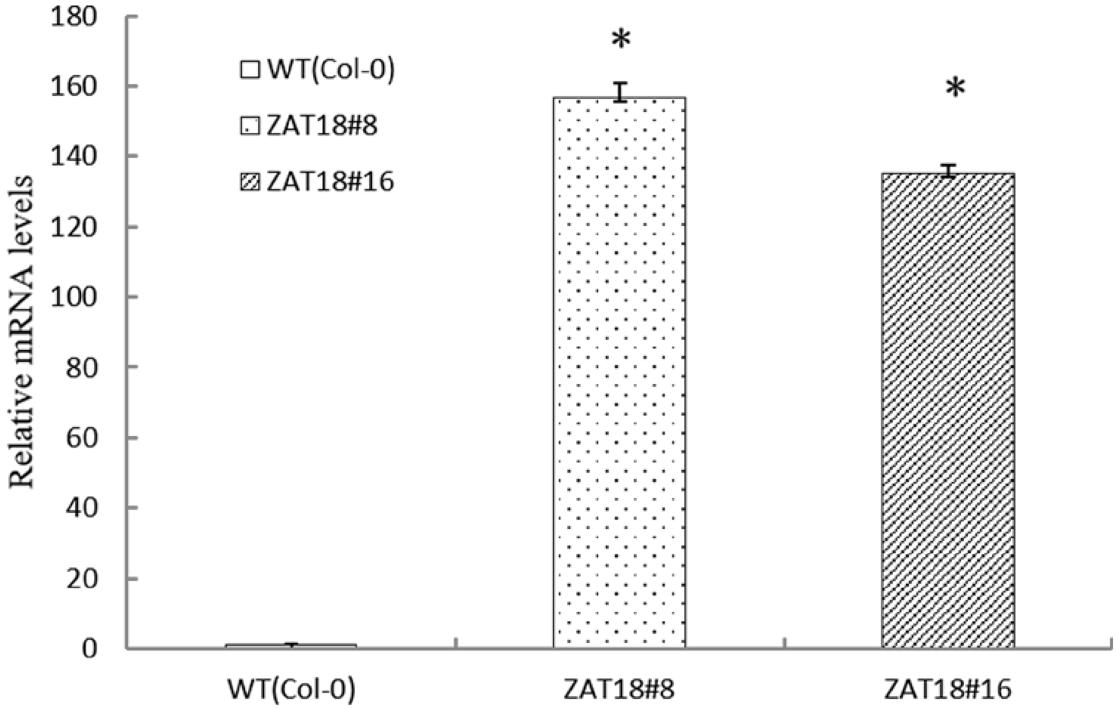

Supplementary Fig.2. Expressing level changes of ZAT18 target genes using qRT-PCR

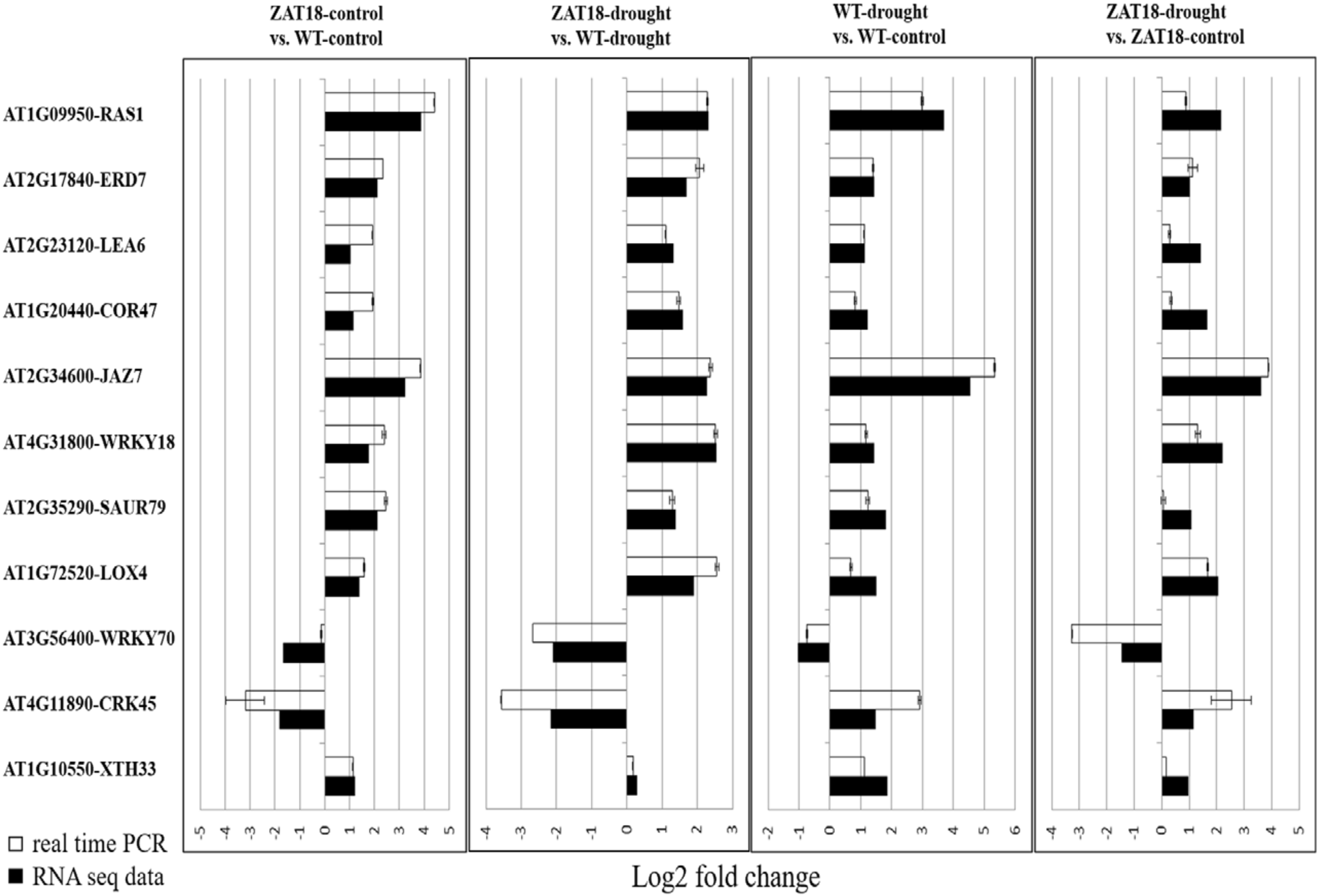

Supplementary Table 1. The primers used for plasmid construction and identification of SALK _027144C and SALK_132289C in this study.

Primer	Sequence(5’-3’)
ZAT18-F	TCTAGAATGAAGAGAGACCGGTCCGA
ZAT18 -R	GGTACCATTCACAAACTTCAAATCAATTTG
ZAT18Promoter-pGUS-SalI-HF-F	ACGCGTCGACGGTTCGATGGTAACATTAAG
ZAT18Promoter-pGUS-XmaI-R	TCCCCCCGGGGTTTTTACGGTTGTATTTCG
ZAT18-RT-F	CAGGTGAAGCATCGTAACAAAG
ZAT18-RT-R	AACCACAGAAGGGACAATCG
UBQ10F	TCCGGATCAGCAGAGGCTTA
UBQ10R	TCAGAACTCTCCACCTCAAG
ZAT18-LB	ATTTTGCCGATTTCGGAAC
SALK_132289C -LP	ATCGATTGCTCGGTTATCATG
SALK_132289C -RP	TCACGATTGACAGTCCAAATG
SALK_027144C -LP	ATCGATTGCTCGGTTATCATG
SALK_027144C -RP	TTTAAACGCAGTCACGATTCC

Supplementary Table 4. The primers of ZAT18 candidate target genes used for qRT-PCR

Primer	Sequence(5'-3')
RAS1-RT-F	CACCAGAAAGACCGGATCTC
RAS1-RT-R	TTTAAGCACCTCCATCGCC
ERD7-RT-F	AAGTTCACCCTGACACCTTG
ERD7-RT-R	ATCCGTCAAGAGATGCAAGG
LEA6-RT-F	GAAAACGGAGCAAGTGAAAGAC
LEA6-RT-R	AGAAGGAGTAGGAGCATCGG
COR47-RT-F	AGAACAAGCCTAGTGTCATCG
COR47-RT-R	GTCCTTTCTTATCTTCCTCTCCTTC
JAZ7-RT-F	CTTATGATTCTGATTTCCATAGCTCG
JAZ7-RT-R	CTCTTCCACATCTCTACTCGC
WRKY18-RT-F	TCAACACCAATCCTTTCTCCG
WRKY18-RT-R	TGTAGCTCTCACAGACTCTAGC
SAUR79-RT-F	TCCTGTAATGAAACTCCGTCG
SAUR79-RT-R	CACGATCCTTAGCTGAACCTATC
LOX4-RT-F	TGGCGGTTTGATATGGAAGG
LOX4-RT-R	GTTGGCGTATGGGTAGTCTTC
WRKY70-RT-F	GGGATCTCAAAATGCTTCATGTG
WRKY70-RT-R	CCAAGAAAATGCGTCCTCAAG
CRK45-RT-F	CGTGGCGGATTTGGTTTTG
CRK45-RT-R	CATGTTGGTCTCGTTTAGTGC
XTH33-RT-F	ATATTCAGGTCAATGGCTCCC
XTH33-RT-R	TCGGATAAGTCTCTGCGTTTG
